# Why People Drink Shampoo? Food Imitating Products Are Fooling Brains and Endangering Consumers for Marketing Purposes

**DOI:** 10.1371/journal.pone.0100368

**Published:** 2014-09-10

**Authors:** Frédéric Basso, Philippe Robert-Demontrond, Maryvonne Hayek, Jean-Luc Anton, Bruno Nazarian, Muriel Roth, Olivier Oullier

**Affiliations:** Psychology@LSE, London School of Economics and Political Science, St Clements Building, London, United Kingdom; 2 Graduate School of Management, University Rennes 1 & Center for Research in Economics and Management, UMR CNRS 6211, Rennes, France; 3 Aix Marseille Université, CNRS, LPC UMR 7290, Cognitive Psychology Laboratory, Fédération de Recherche 3C, FR 3512, Case D, Marseille, France; 4 Poison Control Centre, Hôpital Salvator, Marseille, France; 5 Centre d’IRM Fonctionnelle Cérébrale, Institut de Neurosciences de la Timone, UMR 7289, Aix-Marseille Université, CNRS, Marseille, France; University of Texas Health Science Center at San Antonio, Research Imaging Institute, United States of America

## Abstract

A Food Imitating Product (FIP) is a household cleaner or a personal care product that exhibits food attributes in order to enrich consumption experience. As revealed by many cases worldwide, such a marketing strategy led to unintentional self-poisonings and deaths. FIPs therefore constitute a very serious health and public policy issue. To understand why FIPs are a threat, we first conducted a qualitative analysis on real-life cases of household cleaners and personal care products-related phone calls at a poison control center followed by a behavioral experiment. Unintentional self-poisoning in the home following the accidental ingestion of a hygiene product by a healthy adult is very likely to result from these products being packaged like foodstuffs. Our hypothesis is that FIPs are non-verbal food metaphors that could fool the brain of consumers. We therefore conducted a subsequent functional neuroimaging (fMRI) experiment that revealed how visual processing of FIPs leads to cortical taste inferences. Considered in the grounded cognition perspective, the results of our studies reveal that healthy adults can unintentionally categorize a personal care product as something edible when a food-like package is employed to market nonedible and/or dangerous products. Our methodology combining field (qualitative) and laboratory (behavioral and functional neuroimaging) findings could be of particular relevance for policy makers, as it can help screening products prior to their market release – e.g. the way they are packaged and how they can potentially confuse the mind of consumers – and therefore save lives.

## Introduction

In 2006, *ConsumerReports.org* – a website edited by Consumers Union, an organization based in the United States – exposed that *“some cleaners look like beverages”* and led to numerous cases of poisoning in the home [1]. This was revealed by a study conducted at the Texas Poison Control Centre [2]. From January to April of 2006, a surprisingly high volume of calls reported an unusual series of accidents related to a specific household cleaner known as *Fabuloso*. As many as 94 unintentional ingestions occurred, 39 of which implied healthy adults. Unfortunately, these cases were not specific to the US market. In Europe, accidental ingestions (some with lethal outcomes) were also reported in 2011 [3]. The repeated cases led for this public health issue to be named and now to be referred to as Food Imitating Products (FIPs). FIPs are defined by article #1 of a legislative act of the European Union – European Council Directive 87/357/EEC [4] – as products that “[…] *although not foodstuffs, possess a form, odour, colour, appearance, packaging, labeling, volume or size, such that it is likely that consumers, especially children, will confuse them with foodstuffs and in consequence place them in their mouths, or suck or ingest them, which might be dangerous and cause, for example, suffocation, poisoning, or the perforation or obstruction of the digestive tract*” [4].

Subsequently, in 2001, the General Product Safety Directive (2001/95/EC), a key legal framework for the European common market, launched the RAPEX, a rapid alert system for non-food consumer products [5]. The RAPEX enforces the withdrawal of all FIPs from the European market and to *“restrict the marketing or use of consumer products posing a serious risk to the health and safety of consumers”* ([6], p.12). Marketing, especially product marketing, seems to be a key factor in confusing consumers and leading not only for children to be poisoned [7] but to fooling healthy adults as well. Regarding *Fabuloso* – the floor cleaning product mentioned earlier that caused so many poisoning cases in Texas – physicians from the State of New York pointed that: “*ultra-modern designs of Liquid Cleaner Agents* [LCA] *in order to make them more attractive* […] *may be a source of unintentional exposures in children and adults*” ([8], p.745).

Product package is well known to contribute to the experiential aspects of consumption (fantasies, feelings and fun). The act of consuming is no longer to be reduced to a utilitarian perspective: consumption has become hedonic [9]. Regardless the risks for the health of the consumers, some product managers openly disclose in the press that people should no longer be ashamed to display hygiene products in their homes [10]. Anchoring product package in “food imaginary” is a differentiation strategy that intends to minimize the lack of commercial appeal of some products, the burden they represent (cleaning the house) and their potential danger [11]. This is everything but surprising in light of findings supporting strong links between food and subjective (hedonic) experience of consumption at the neural [12], conceptual [13], [14] and social levels [15].

In the present series of studies – conducted on the field and in the lab –, we propose to consider the food imaginary associated with the package of FIPs in the framework provided by the conceptual metaphor theory [16]. Indeed, “*marketers contribute to the stock of marketplace metaphors through both packaging and advertising*” ([17], p.232). The conceptual metaphor theory therefore seems to be a good candidate for the investigation of the rationale behind the marketing of FIPs. Moreover, this theory has proven useful to analyze and better understand public health related issues such as tobacco (e.g., [18]), medicalization [19], physician/patient relationship [20], epidemics [21], how packages are designed [22] and even current marketing strategies (e.g., [23]–[36]).

The essence of a metaphor is to experience one kind of thing in the terms of another [16]. In the case of FIPs, a (novel and non-verbal) metaphor such as: hygiene products are food [37], draws the attention of people purchasing household cleaners to an appealing and somewhat positive aspect of the product while masking their more negative and sometimes dangerous features. The experience of the source domain (food) is mapped onto the target domain (hygiene products) [38]. Thanks to the implicit (gustatory) inference at the core of a (food) metaphor [39], such a strategy intends to further the brain of the consumer from some attributes of the product that could not be commercially appealing. The concept of hygiene products, and the not so entertaining and hedonic baggage that comes with it at the aesthetics and behavioral levels (sweeping, cleaning, etc.), is intentionally masked by the manufacturers’ use of metaphors associated with food, the latter being way more appealing and pleasurable than hygiene products. Hence, in the case of FIPs, the consumer will “forget” how boring and unattractive the imaginary of a hygiene product can be as (s)he is manipulated to non-consciously focus on more exciting attributes associated with the pleasure of eating.

We therefore entertain the idea that metaphors – and their sensorimotor origin – guide behaviors: they could influence our experiences and our actions [16], leading to accidental poisoning in the home in the case of FIPs. Furthermore, one might be able to identify (the effects of) food metaphors at the cerebral level [39]–[41] because of the possible increased activity in gustatory cortices of the brain they might elicit. However, according to the epidemiologic *host-agent-environment* triad [42], [43], consumers’ self-poisonings could be related to individual (host) or contextual (environment) influences rather than solely to the appearance of the product *per se* (agent) and its marketing strategies.

Therefore, many questions regarding FIPs are still pending as they are under investigated worldwide, in spite of the European Council’s Directive 87/357/EEC [4] and cases like those reported in Texas [2] and in the State of New York [8]. The European Commission’s (EC) Scientific Committee on Consumer Safety (SCCS) points at the lack of (scientific) studies on FIPs in both lab and field settings recommending to utilize “*the information collected by poison centers* [that] *are required in order to identify product groups associated with the highest risk of poisoning and to describe the circumstances that lead to oral intake*” ([44], p.2).

This is why, to understand the rationale behind the dangerous marketing strategy that FIPs represent and the related risks for the health of consumers [7], we decided to start with a field study on real life cases of home poisoning. The goal of this first step was to identify some dangerous products that are available on the market. This was followed by laboratory experiments to test at the behavioral and brain levels the effects of some of the FIPs identified during the qualitative field study. To our knowledge this is the first time in consumer behavior studies that intra- and inter-individual multilevel research is conducted articulating qualitative (physician-patients conversations) and lab (behavioral and neuroimaging) studies and findings. By considering real products involved in home poisoning cases with real people, we differ clearly from most lab studies in consumer neuroscience and neuromarketing that very often test highly artificial products that often do not exist in real retail settings [45] but are always considered outside the *host-agent-environment* interactions [42], [43].

Our work introduces a new way to investigate public health issues by combining real life information sourced on a large-scale sample together with behavioral experiments and neuroscientific insights collected in the laboratory context. Our goal is to shed new light on the behavioral and neural underpinning of the confusion operating in the minds of consumers because of the marketing strategy behind FIPs. This research could help develop a new procedure to test FIPs prior to their launch on the market but could be used in many other domains, beyond the FIP context. In light of our previous work with national and supra-national governing bodies advocating for using behavioral and brain insights to inform policy [37], [46], our results and our procedure could therefore be of particular relevance in the public health policy sphere. For instance, it could be considered in the General Product Safety Directive [5] – currently under revision and expected to come into effect in 2015 [47] – and/or by the call for measure to protect consumers against FIPs expressed by a European online consumer education platform [48] and by associations for consumers’ protection (e.g. US Consumer Union in 2006 [1] and 2012 [49]).

## Overview of the Studies

In order to test for potential gustatory inferences triggered by FIPs, we first conducted a qualitative field study on more than 30,000 phone calls made to the Marseille Poison Control Center over a period of 14 months [37]. It allowed to select a product (FIP) that used a food metaphor in its package and had been accidentally ingested by healthy individuals in real life, rather than picking up one independent of its purchase and/or consumption context as it is too often the case in consumer neuroscience and neuromarketing studies. The appetitive dimension of this product was first evaluated against other FIPs and non-FIPs at the behavioral level. Our main hypothesis was that implicit gustatory inferences – i.e. neural activations in gustatory cortices – will be observed when consumers look at metaphorical hygiene products (compared to non metaphorical ones). An experiment using functional magnetic resonance imaging (fMRI) was then conducted. Comparisons of gustatory inferences at the cortical level were made between a FIP, a non metaphorical accidentally ingested hygiene product, a fruit juice and a ‘classically shaped’ bottle of bleach (the last two serving as controls). As we were working on the neural correlates of processing visual food cues, prior to scanning brains, we made specific controls on participants (Body Mass Index (BMI), fasted time, food preferences), stimuli (valence and arousal) and neuroimaging data acquisition settings.

All together, these qualitative and laboratory studies are meant to provide new information regarding the non-conscious neural processes elicited by the FIP and how they could participate in the confusion occurring in the mind of the consumer, possibly leading to poisoning in the home.

## Study 1: Qualitative Analysis of the Data Collected on the Field

The need for running our own qualitative study on medical cases is related to the lack of homogeneity and accuracy of existing medical data on FIPs. The qualitative analysis allowed to better investigate and understand the circumstances under which poisonings in the home by accidental ingestion of hygiene products occurred.

Funded by the European Chemical Industry Council (CEFIC), a large scale study was conducted by the DeNaMiC project (Description of the Nature of Accidental Misuse of Chemicals and Chemical Products) to determine the availability of information from poison control centers and other sources and to characterize the nature of accidental exposures to these products to inform and improve risk management [50]. This study revealed the lack of homogeneity of the data on poisonings related to household chemical products. As highlighted by a UK Health Protection Agency publication: “*Published statistical data and literature analysis on the nature and frequency of incidents and events related to accidental exposures of chemical products could not be analyzed statistically due to the heterogeneity of the data. The information commonly reported varied dramatically due to a lack of a standard reporting format*” ([51], p.43). Similarly, another study by the DeNaMiC project concluded that publicly available data on accidental exposures to chemical products provided little on the circumstances of exposure and could only be compared qualitatively [52]. It seems that location, use and storage of household chemical products by the consumers are not documented routinely in all poison control center databases [53].

Moreover, because of the turn over and renewal of products on the marketplace, and the extent to which the name of various products vary from one country to another, poison control center databases collect information on product categories (e.g., bleach, disinfectants, cosmetics, detergents, etc. [50]) rather than on products’ brands and names. However, this information is crucial when one wants to check whether a hygiene product accidentally ingested is a FIP, at the national and/or international levels, and, when possible, take the appropriate measures to withdraw it from the market to protect the lives of the consumers.

In order to improve both the homogeneity and the accuracy of the data on FIPs, we therefore conducted a qualitative study on naturally occurring talks [54] (physician-patient phone calls) selected from a poison control center database. The COREQ guidelines were followed for reporting this study [55].

### Methods

#### Ethics statement

All participants were informed that the phone call they placed to report the accident of interest was recorded. The written transcripts of the physician-patient phone calls were anonymized and their use in the study received the approval of local (Aix-Marseille Université Ethics Committee), regional (Comité de Protection des Personnes Sud Méditerranée 1) and national ethics and regulatory agencies (French Agency for the Safety of Health Products/Agence Française de Sécurité Sanitaire des Produits de Santé) – this was the case for all the studies reported in this article.

#### Participants and setting

To select cases of poisoning related to our topic of interest (FIPs), we examined medical records received at the Marseille Poison Control Center (MPCC).

The MPCC is part of French Public Hospital System (Assistance Publique – Hôpitaux de Marseille, France). Phone calls placed to the MPCC therefore constitute medical cases *per se* for investigative epidemiology [56]. We were interested in cases that report accidental poisoning in the home when a healthy adult would ingest a hygiene product that was mistaken for a food beverage. For information purposes, when a call was placed by someone else than the patient – due to the fact that (s)he was unable to talk given that (s)he drank a household cleaner or a body care product – we only kept the case for further analysis if the caller were physically present with the patient (additional details are provided in the following sections on data collection and analyses).

#### Data collection

Due to legal constraints, all phone calls placed to the MPCC 24-hour emergency phone unit were recorded and kept in a medical file. Data was then stored in the national database of products and composition (BNPC) thanks to the poison control center information system (SICAP). When searching this database, it is possible to find all the medical files related to one kind of poisoning and/or product. The medical files detail the patient’s name and surname, gender, age, weight, home address, phone number, medical history and medical treatment as well as the date, time, place and circumstances under which the poisoning occurred in addition to the incriminated product name and medical monitoring.

To select the poisoning cases that will undergo our qualitative analysis, a specific item – named “confusion between hygiene product and food” – was created and added to the MPCC checklist and database. The local medical staff was therefore asked to rate this item for each poisoning case meeting this criterion. Because of interpersonal differences among medical staff members in compliance with item rating requirement, a careful and detailed examination of each of the 31,283 medical records collected over the 14-month period at MPPC was also performed.

Data was first coded as a medical case by MPCC physicians and, subsequently, a detailed examination of each of the medical records was performed including listening again to the phone call recordings and re-coding the data (e.g. audio-to-text transcription), when necessary.

Given the call volume and our qualitative methodology, we decided to limit our selection of poisoning cases to (1) ingestion of (2) products that can be identified (brand and/or name) (3) occurring at home by (4) healthy (5) adults.

We first selected 515 medical cases in which adult patients ingested a hygiene product. Among these 515 medical cases, we kept the 477 related to healthy individuals. Among these 477 medical cases, 465 accidental ingestions took place at home. Finally, only 44 of these 465 medical records provided clear information of the ingested product (brand and/or name).

Our choices limit dramatically the number of calls to undergo the qualitative analysis but are justified by our willingness to avoid cases of misuse (e.g. spatter instead of ingestion or even cases where the content of a product is put in another package), occupational injuries (most of professional products are not aestheticized and are not of particular relevance to the usual consumer in his/her house), intentional poisoning (murder attempts, psychiatric or suicide cases, the latter being the first cause of caustic ingestions in adults [57]) and ingestions by children.

We concur with Kopjar and Wickizer who emphasized the interest of paying attention to *“the problem of unintentional home injuries among adults and to the development of appropriate prevention strategies”* ([58], p.403).

Notwithstanding, our decision to exclude cases involving children deserves a little more explanation, especially since they constitute a significant call volume and an important area of research [59], [60]. First, we were interested in naturally occurring talk about the reasons and circumstances why poisoning occurred. This is why we never phoned the callers and/or the patients back (physician-patient interactions were spontaneous, one-shot and no transcripts had been returned to patients for comments or correction). Second, given the Haddon matrix [61] applied to risk factors for children injuries [62], we know that these cases often take place in the absence of parental supervision and that children are unable -or unwilling- to communicate accurately about the accident (for example, some cannot even talk or some might lie to avoid upsetting their parents). Hence, even without considering the natural curiosity of children [63], the real circumstances why and how the poisoning occurred generally would be very likely to remain unknown. Then, if we bear in mind the specificity of any research involving children [64], we would like to suggest that if a metaphorized product can lead to adult unintentional poisonings, *ipso facto* it can lead to children poisonings as well. This is why, even if the ingestion of hygiene products by adults remains a small part of the call volume to poison control centers, it enables to understand what most exposed and poisoned people themselves, i.e. children, cannot describe.

#### Data analysis

Our qualitative study is a content analysis of the naturally occurring talks collected both by FB (PhD) as a social scientist who was not affiliated with the poison control center and its staff and MH (MD) as the head of the MPCC. These two researchers had no relationship established with the participants (patients and callers). These naturally occurring talks have been transcribed by FB.

On the basis of the content analysis of these naturally occurring talks, we looked for 8 attributes listed in the first article of the European Council’s Directive 87/357/EEC [4] related to FIPs: form, odor, color, appearance, packaging, labeling, volume or size. Indeed, these criteria are very close to the specific elements that clearly contribute to the metaphoric meaning of a hygiene product that is available on the market (verbal prose, visual images, shape, color and scent) and were also identified by Hirschman [17]. Metaphors induce a specific way of thinking and acting towards a product when used as a marketing strategy. Applied to hygiene products, a food metaphor can lead to an action that is not cleaning or sweeping *per se* but ingesting a cleaner or a body care product. Something that, from a health (policy) perspective, is not acceptable.

### Findings

The naturally occurring talks reported are the 20 explicit cases illustrating the effects of non-verbal food metaphor in accidental poisoning of healthy adults (see Table 1 and Table 2).

**Table 1:** General information about the poisoning cases analysed in the qualitative study.

Medicalcase #	Gender^1^	Age	Caller	Product type	Brand and/or name of the ingested product
A	F	69	Husband	Household cleaner(dish liquid)	*Visior* sweet almond extract
B	M	21	Friend	Household cleaner(bleach tablet)	Bleach tablet
C	F	37	Physician	Household cleaner(dish liquid)	*Visior* hygiene plus
D	F	30	Friend	Personal careproduct (shampoo)	*Champion* henna and hazelnuts for brunettes
E	F	77	Patient herself	Household cleaner(softener)	*Soupline* lavender
F	F	41	Husband	Personal careproduct (shower gel)	Cottage Happy Shower Tequila Sunrise
G	F	72	Emergencyphysician	Household cleaner(multi-purpose)	*Tropic force* multi-purpose household cleaner fruity atmosphere
H	M	>50	Wife	Personal careproduct (liquid soap)	*Le Comptoir de Famille*tomato leaf liquid soap
I	M	80	Emergencyphysicianthenpatient himself	Household cleaner(dish liquid)	*Mir* vaisselle
J	F	54	Friend	Household cleaner(ironing liquid)	*Cajoline Vaporesse*
K	M	40	Emergency physician	Household cleaner(multi-purpose)	*Milodor* strawberry
L	F	72	Patient herself	Household cleaner(parquet floor cleaner)	*O’Cedar* for stratified andlaminated flooringmodern parquet
M	M	85	Nurse	Personal careproduct(shower gel)	*Adidas* Sport Field menthol
N	F	76	Husband	Personal careproduct (cleansing)	*Eau Précieuse*
O	M	>50	Patient himself	Household cleaner(dishwasher tablet)	*Skip Actigel*
P	F	21	Emergency physician	Personal careproduct (slimming cream)	*Cosmence*
Q	F	83	Friend	Household cleaner(dish liquid)	*Apta* berry vinegar
R	M	23	Patient himself	Personal careproduct (hair gel)	*Schwarzkopf Got2b*
S	M	89	Son-in-law	Household cleaner(dishwasher tablet)	Dishwasher tablet
T	F	60	Mother	Householdcleaner (softener)	*Soupline*

1 M: Male; F: Female.

**Table 2:** Excerpts of 20 illustrative poisoning cases recorded at the Marseille Poison Control Center (transcript from the MPCC audio recordings and French-to-English translation by the authors).

Medicalcase #	Poisoning description	Naturally occurring talk – Physician-patient phone calls
A	*Visior* sweet almondextract dish liquidpoured like syrup^1^.	- Caller: […] *My wife made a mistake, instead of taking the bottle of mint syrup* 1, *she took the bottle we use to wash the dishes, they have the same color.* [….] *A sweet almond flavor* […] *I hand my wife the phone*.- Patient: *I poured very little of it. I thought I was pouring mint, it’s the same color and both bottles are next to each other under the sink. I took that and I poured a little bit. I just poured it then I filled my glass with water.*
B	Bleach tablet mistakenfor a candy.	- Caller: *Hello I’m calling because there is someone besides me who just put a bleach tablet in his mouth.* […]- Physician: *It’s a mistake? An error?*- Caller: *He thought it was a candy*.
C	*Visior* dish liquidpoured like syrup^1^.	- Caller: […] *She thought it was mint syrup* 1, *she was still a bit asleep. In addition, the product is blue. I asked her to bring the product. Her husband went to pick it up.* […]- Physician: *Do you know if it has been diluted?*- Caller: *Yes, it was diluted. She put some in the glass similarly to the way you would put syrup* 1 *before mixing it with water* […].
D	*Champion* henna andhazelnuts forbrunettesserved as honey.	- Caller: *Good afternoon, Madam*, […] *my girlfriend swallowed a mouthful of shampoo looking like honey*. […]- Physician: *She thought it was honey, right?*- Caller: *Yes. The pot is exactly the same.*
E	Mouthful of *Soupline*lavender softener.	- Patient: *Madam, here what happening to me. I thought I took a bit of water and the bottle happened not to be in its usual spot, and I swallowed a mouthful of Soupline, you know what one for rinsing. So I tried to make myself throw up. I called because I’m wondering what I have to do*. […]
F	*Cottage Happy Shower Tequila Sunrise*shower gel mistakenfor an orange juice.	- Caller: *Hello Sir, I call you because my wife just swallowed a mouthful, it’s a shower gel. Let me explain why she has ingested some. Because it is a bottle that is… I just came back from grocery shopping and there is this bottle that looks like a bottle of fruit juice, green with orange, but ultimately it is …* [He mumbles reading a description on the product.] *What is that? Yeah, it’s a shower gel. So inadvertently, she believed that it was orange juice. She took it and she has swallowed a mouthful.* […]- Physician: *OK. It’s some orange flavor. It was a tube, well, how could I explain…*- Caller: *Well, it is a new product that’s why… It looks like a bottle, the capacity is 250* *ml, flashy green. It may just be a bottle of orange juice. In addition, the cap is like the bottle caps … the ones you pull.*- Physician: *Yeah. A bit like one of those bike things*.- Caller: *Absolutely. It is true that this leads to confusion. One should be well advised to paying attention to this because … even for children and everything.*- Physician: *Exactly. That’s why I ask for clarification* …- Caller: *Yes, I have seen others like this in the alley of the supermarket, but ultimately it’s true that if I had seen it myself on a table, I see orange slices, I would have grabbed it…*
G	*Tropic force* multi-purpose householdcleaner fruityatmosphere mistakenfor a drink.	- Caller: *Hello, this is Dr. X from the Cayenne E.R. I’m calling about a woman, 72 years old, who, earlier, about a half-hour ago, drank some household cleaner* […]. *It smells very fruity and it was stored in the freezer.*
H	*Le Comptoir de* *Famille* tomato leafliquid soap mistook forcough syrup^1^.	- Caller: […] *My husband had to take some cough syrup* 1, *the Surbronc one, and he made a mistake – not paying attention – there was, close to the sink, some soap but a liquid one with a tomato leaf flavor. So I’d like to know…*- Physician: *… he took a soup spoon isn’t it?*- Caller: *No. A coffee spoon.*
I	*Mir* vaisselle dish liquidpoured like muscatel.	- Physician: *So you drank a bit of Mir dish liquid?* […] *That is? You thought it was syrup* 1 *or it was some leftover in a glass?*- Patient: *Excuse me?*- Physician: *How was it? You took it for syrup* 1 *by yourself or was there some leftover in a glass…*- Patient: …*for muscatel wine*!- Physician: *For muscatel wine! Well, well!*- Patient: *Same color.*
J	*Cajoline Vaporesse*ironing liquid mistakenfor water.	- Caller: *Good evening. Poison center?*- Physician: *Yes.*- Caller: *I call you from Corsica. There is a woman who was ironing at my home. She wanted to drink a and she made a mistake between two bottles.*- Physician: *What did she drink?*- Caller: *She drank some Cajoline the one that you pour in your iron. She took a sip.* […]
K	*Milodor* strawberry multi-purpose cleaner pouredlike syrup^1^.	- Caller: *It’s some kind of thing stuff, yes. In fact it’s something that reads in essence: "Strawberry" it’s all red and he mistook it for syrup* 1.
L	*O’Cedar* parquet floor cleaner mistaken for a juice fruit.	- Patient: *It’s a very pretty pink liquid that looked like my bottle of Guava.* […] *I drank from the bottle as I always do. Hence I must have swallowed a good mouthful.*
M	*Adidas* Sport Fieldmenthol shower gelpoured in a glass.	- Caller: *Good morning. I am a nurse in a nursing home in Montpellier. Look it’s perhaps nothing but one of my residents has a priori drunk a shower stuff called Adidas Sport Field menthol*. […]
N	*Eau Précieuse* 2 cleansingmistaken for water.	- Caller: *Hello, I call you about my wife.* […] *She takes tablet of caffeine-Propofan because she has a headache. She took the wrong bottle, instead of taking a bottle of water, she had some sips of Eau Précieuse* 2 *the one for cleaning the face. I would like to know if it’s dangerous, if there is something to do.*
O	Skip Actigel diswashertablet mistaken for acookie.	- Patient: *Good evening. I just called because I thought it was a cookie and …*- Physician: *… you have taken a bite of a dishwasher tablet haven’t you?* 3- Patient: *Yes.* […] *I was watching a game on TV and I wanted to eat something, my wife is so clever, she put it with cookies we buy for our grandchildren.*
P	*Cosmence* slimmingcream ingested.	- Caller: *Good morning, this is Dr X. from Porto-Vecchio. I call you because of a young lady that made a mistake. There was a product for losing weight to apply on the skin and actually she diluted it in water and she drunk it all day long* […]
Q	*Apta* berry vinegar dishliquid mistaken forvinegar.	- Caller: *So I have my friend who has ingested a little bit, because she tasted a sauce, a sauce which she took, she took it for vinegar, she ate detergent.*[…] *You know what they wrote below, they put* “berry vinegar!”
R	*Schwarzkopf Got2b* hairgel mistaken formayonnaise.	- Patient: *I’m calling because, in fact, you are going to laugh, but I actually screwed things up and took a tube of hair gel for a mayonnaise one. The gel is one of those yellow ones, I do not know if you see what I’m talking about?* […]- Physician: *Okay. So how much of it have you ingested?*- Patient: *Actually I put some on top of a tomato, then the equivalent of what … a finger.*
S	Dishwasher tabletmistaken for nougat.	- Caller: *My stepfather, who is 89, has taken a dishwasher tablet thinking it was nougat, and he swallowed but not much.*
T	Sip of *Soupline* softener.	- Caller: *Hello, good morning Madam, I am on vacation in Aude and my daughter inadvertently had a sip of* Soupline, *is it dangerous?*- Physician: *How old is your daughter?*- Caller: *well… 60 years old!*- Physician: *… and therefore the* Soupline *was poured in what*?- Caller: *She was distracted. She was cleaning the table, she let the stuff, she thought it was…*- Physician: *… and she didn’t have look at the bottle at all, she took it directly…*- Caller: *She took a glass*.- Physician: *Therefore it was in the* Soupline *package, do we agree?*- Caller: *Yes, yes, yes.*- Physician: *Well. Because sometimes one pours it in food bottle and this leads to even more mistakes.*- Caller: *No no no. She did not pay attention. I laugh but it’s not funny.* […]

1 The reader has to be informed that in the conversations we report “syrup” refers to some thick sugary liquid that people pour in an empty glass and then mix with water (generally one volume of syrup diluted with 6 volumes of water). In France they are known as “sirops” and are very popular. Among the most popular are “sirop de menthe”, literally “mint syrup” thick and green or blue, or strawberry syrup. Hence, in the context of this article syrup does not refer to something like maple syrup that can be found on the American market for example.

2 The reader has to be informed that the “Eau Précieuse” cleansing referred in this conversation literally means “precious water”. This is why labeling seems to be key in this accidental ingestion.

3 The MPCC physician was primarily informed by a physician working for the French equivalent to 911.

As reported in Table 3, all of these hygiene products met at least one of FIP criteria – to the notable exception of one call in which no information that could relate to these criteria whatsoever regarding the product was available (Soupline lavender #E).

**Table 3:** FIP criteria (as proposed by the European Commission) met by each of the products cited in the MPCC calls reported in previous tables.

Medical case#	Brand and/or product name	Form^1^	Odor	Color	Appearance	Packaging	Labeling	Volume	Size
A	*Visior* sweet almond extract		x	x			x		
B	Bleach tablet				x				
C	*Visior* hygiene plus			x					
D	*Champion* henna and hazelnutsfor brunettes					x			
E	*Soupline* lavender								
F	*Cottage Happy Shower*Tequila Sunrise	x	x	x		x	x	x	
G	*Tropic force* multi-purposehousehold cleaner fruity atmosphere		x				x		
H	*Le Comptoir de Famille* tomato leaf liquid soap						x		
I	*Mir* vaisselle			x					
J	*Cajoline Vaporesse*					x			
K	*Milodor* strawberry			x			x		
L	*O’Cedar* stratified andlaminated flooring modern parquet			x		x			
M	*Adidas* Sport Field menthol						x		
N	*Eau Précieuse*					x	x		
O	*Skip Actigel*				x				
P	*Cosmence*				x				
Q	*Apta* berry vinegar						x		
R	*Schwarzkopf Got2b*	x		x					
S	Dishwasher tablet				x				
T	*Soupline*					x			
**Total**		2	3	7	4	6	8	1	0

1 This table provides the coding of each product’s attributes, both listed in the FIP Directive [4] criteria and referred by a patient (or a caller) in explaining his/her accidental poisoning ingestion. Form, color, odor, volume and size are criteria easier to code than appearance and packaging, given the difference between product appearance and product packaging is not obvious (e.g., [139]). In this table, we have coded “appearance” as confusing attribute only for solid products (e.g., bleach tablet, dishwasher tablet, etc.) and “packaging” only for liquid products (e.g., shower gel, shampoo, etc.). Regarding to labeling, this criteria is fulfilled each time the product or brand name is confusing (e.g., Eau Précieuse (#N) given it means “precious water”).

Three main conclusions can be drawn from this qualitative analysis.

First, there are more liquid hygiene products confused with food (beverages) than solid ones. However, we have to bear in mind that this could be related to the choices we made by selecting only branded products, given that solid hygiene products (such as dishwasher or washing machine tablets) are not easy to name once they are out of their initial package.

Second, labeling, packaging and color are the visual dimensions that are cited the most by patients (or callers). But even if form, volume and/or size are rarely reported as confusing factors, they should not be neglected. Poisonings happen because products can be manipulated. For instance, a 5-liter bottle is not likely to be confused with a strawberry syrup bottle that usually has a capacity of 0.75 to 1 liter. In addition, there is little mention of odor in phone calls. If the smell of a product were unpleasant, consumers would not have drunk these hygiene products. By essence, a hygiene product is meant to smell good – but may be not ‘too good’ to avoid its ingestion.

Third, the attributes of products are probably related to these accidental poisonings as stated by patients (or callers). But, as emphasized in many public health studies, the role of contextual and personal factors should not be underestimated in an accidental exposure to a toxic agent [42], [43]. Studies in (consumer) psychology clearly show how contextual and personal goals can modify the way people categorize products [65]. This means that hygiene products could have been miscategorized as food or beverages not only because of their attributes but also because of the context and/or patients’ goals. Regarding the latter, findings from qualitative study reveal that hygiene products had been ingested while patients were looking for a drink or wanted to take some medicine. Hence, one cannot rule out the possibility that, in poisoning cases, FIPs neither prevented confusion, nor did they create it. Regarding the contextual factor, results remain somewhat unclear. Some of the FIPs listed in Tables 1, 2 and 3 have been ingested while they were stored out of their context of use (e.g., Tropic force #G, Skip Actigel #O) but some others have not (e.g., Comptoir de Famille tomato leaf soap #H, Apta berry vinegar #Q).

Taken together, these findings do not allow us to conclude that each of the FIPs selected are dangerous on their own. However, as raised by Norman: “*If an error is possible, someone will make it*” ([66], p.36). Or, as he also put it: “*Most accidents are attributed to human error, but almost in all cases the human error was the direct result of poor design*” ([66], p.viii). In this article we therefore adopt a system approach rather than a person approach of human error [67]. In order to show that the accidental ingestions of FIPs are not only related to the context and the goals of consumers, we propose to test whether some of the products identified in our qualitative study and involved in accidental poisoning could lead to a confusion in an experimental setting where these two factors (context and goals) are controlled.

## Study 2: Behavioral and Functional Neuroimaging Experiments Conducted in the Laboratory

In light of the evidence and the limits of the qualitative study, we decided to test whether the food metaphor applied to a hygiene product could lead to implicit and automatic gustatory inferences when consumers are looking at such an item. Neuroimaging (functional magnetic resonance imaging; fMRI) was therefore used to estimate brain activity in this particular experimental context. fMRI turns to be of particular relevance when exploring non-conscious (implicit and automatic) processes in consumer research (or else) [68]. For instance, several recent studies have hinted at how neuroscientific insights can provide additional information that classical investigation techniques based on verbalization (surveys, questionnaires, focus group, etc.) cannot [69].

Given that 7 out of 8 EC criteria defining FIPs are visual in nature, we assumed the vision of the product in an experimental setting should be quite informative. A metaphor only makes sense because of the implicit and automatic embodied simulation it affords – also referred to as an *embodied reenactement* [70]–[72]. Grounded in the consumers’ bodily experience of food, these enacted inferences are embodied and have a sensorimotor component with neural counterparts [39]–[41], [73], [74]. In the context of FIPs, this means that we want to know whether a food metaphor applied to a hygiene product leads to increased activity in gustatory cortices, which, of course, is not what a hygiene product should do.

Our hypothesis is that consumers make implicit gustatory inferences (that they should not be making) when looking at hygiene products packaged like foodstuffs and that this should be revealed at the cerebral level in a network of brain activity repeatedly identified when people look at food.

Gustatory inferences are mental simulations. In grounded cognition, mental simulations are generally an automatic and implicit (non-conscious) perceptual reenactment [75] contrary to the simulation already explored in mental imagery (e.g., [76]–[79]). Hence, in the fMRI experiment, we used an experimental paradigm (a 1-back task) where food perceptual simulations (gustatory inferences) that might arise from the metaphorical hygiene product perception were not suggested by the task itself [80]. Moreover, that task ensured that participants allocated their attention to looking closely at the products displayed (e.g., [81]).

A landmark study, conducted in an embodied cognition perspective, showed that food knowledge is distributed across (a network of) modality-specific areas of the brain that participants rely upon when viewing pictures of foodstuffs [80]. The brain areas exhibiting the most significant increase in activity are the primary and the secondary gustatory cortices – the (right) insula/operculum and the (left) orbitofrontal cortex (OFC) – and the fusiform gyrus. Interestingly, this finding is confirmed by an Activation Likelihood Estimation (ALE) meta-analysis on the neural correlates of processing visual food cues [82]. We therefore decided to test whether activity in this brain network significantly increases when people look at a FIP but not when they look at a hygiene product that is not packaged like foodstuffs.

Over the past decade, how metaphors are processed in the brain has been addressed through the combined lens of cognitive linguistics and neuroscience (e.g., [83], [84]). It has been argued, that, given that metaphors can be considered as extra-linguistic knowledge, the cerebral activity associated with their processing and understanding should be localized in the right hemisphere of the brain (e.g. [85], [86]). Yet, many findings from studies on patients and healthy participants (e.g., [87]–[89]), using repetitive transcranial magnetic stimulation (rTMS) [90] or fMRI [91], [92], challenge what appears to be a simplistic view on how the brain would process metaphors (for discussions and experimental demonstrations of the limits of the left-brain versus right-brain hypotheses, see [93], [94]). In light of the inconsistent findings regarding the lateralization of metaphor processing in the brain (see [95]), recent neuroimaging studies have argued for a dependence on context [96] or task demands [97] not only in hemispheric recruitment when the human brain processes metaphors, but also in the cerebral networks underlying this task, as illustrated by a study revealing that textural metaphors activate the somatosensory cortex [98].

However, the key issue in the conceptual metaphor theory is not a matter of lateralization of brain activity but to suggest that any conceptual knowledge is a function of our embodied experience [40], [41]. This is supported by the claim, that there is no longer a theoretical clear-cut distinction between the sensorimotor system and conceptual knowledge [74]. This is why recent neuroimaging embodied cognition studies have shifted their focus towards the relations between perception, action and cognition (e.g., [80], [99]–[101]).

By hypothesizing FIPs are food metaphors, our neuroimaging study focuses on the brain processes at play when the concept of hygiene product is somewhat replaced by the one of food. This is why, in the case of FIPs being understood as non-verbal food metaphors, we decided to focus on studying the automatic and implicit reenactments of embodied food experience resulting from the mapping of one conceptual knowledge (food) onto another one (hygiene product).

### Materials and Methods

#### Ethics statement

All volunteers underwent a mandatory medical exam and gave their written informed consent prior to participating in the neuroimaging exam. The behavioral and functional neuroimaging research protocols received the approval of local (Aix-Marseille Université Ethics Committee), regional (Comité de Protection des Personnes Sud Méditerranée 1) and national (French Agency for the Safety of Health Products/Agence Française de Sécurité Sanitaire des Produits de Santé) ethics and regulatory agencies – as required by the French Bioethics Law following the established guidelines and procedure for running experiments on human participants in this country.

#### Participants

Fourteen healthy adult individuals (8 females and 6 males, age ranging from 20 to 46 years (M = 27.4±8.47; BMI = [18.81–24.48]) volunteered in the experiment which took place at the Marseille Functional MRI Center located in La Timone Hospital. They received a €50 monetary compensation for participating in the experiment. All but one were right-handed, had normal or corrected-to-normal visual acuity and no significant history of medical, psychiatric or neurological illness. Before entering the fMRI scanner, each participant confirmed (s)he fasted for at least 4 hours as required by the experimental protocol, then rated how (s)he liked different drinks (orange juice, apple juice, etc.) on a 7-item Likert scale and performed a brief pre-fMRI scanning task familiarization session. After the fMRI session (i.e. outside the scanner), given the limits of declarative hunger ratings (e.g., [81], [102], [103]) each participant was offered to eat as a behavioral control for hunger. Participants were then debriefed to ensure they identified and categorized visually each of the stimuli properly [104] and that the metaphorical FIP and foodstuff were judged as more appealing than the two other hygiene products.

#### Stimuli selection and preparation

Among the hygiene products selected from our qualitative study, 2 FIPs were of particular interest: a shower gel, the *Cottage Happy Shower Tequila Sunrise* (#F), and a dish liquid, the *Visior* sweet almond (#A).

The first one, the *Cottage Happy Shower*, is interesting on several accounts. First, this shower gel was accidentally ingested at home by a 41-year old healthy woman because of food attributes that meet 6 out of the 8 European Council Directive FIP criteria [4]. Its metaphorical content fits with the elements listed by Hirschman [17]. Visual images are oranges, the shape looks like a bottle of orange juice, the background color of the package is green and it smells like an orange flavor. The verbal prose that can be read on the *Cottage Happy Shower* bottle is explicit: “*Stimulating orange to recharge your batteries… An amazing tequila sunrise scent to get you in the mood. When you wake up in the morning, or before you go in the evening, let the good vibrations take over with Happy Shower and… Feel Good !!”*. Second, the *Cottage Happy Shower* bottle cap is a push-pull one. As the DeNaMiC research project revealed, through the analysis of 457 accidentally ingested products in 5 different European countries: similar push-pull-closures that are used for sports drinks and for liquid chemical products seem to be responsible for many accidents [105]. Third, under the RAPEX system, the authorities ordered the withdrawal of the first version of the *Cottage Happy Shower* from the European market seven months before this French case occurred [106].

The second product, the *Visior* (sweet almond), was accidentally ingested by a 69 year-old healthy woman. Instead of taking a bottle of mint syrup, she took the dish liquid bottle. Except for the color and the labeling (and, to some extent, the odor), the consumer gave no FIP Directive [4] criteria or metaphoric content element. The accident is principally due to the context of the hygiene product: the dish liquid and the mint syrup bottles were next to each other under the sink.

We selected these two hygiene products not only because of the circumstances under which they had been accidentally ingested but also because of the way they were rated on valence and arousal in an ancillary behavioral experiment conducted prior to the neuroimaging one, as recommended for a visual food perception fMRI protocol (*e.g.*, [81]) (see Text S1 for details). Indeed valence and arousal are considered as *“motivational vectors that indicate the degree to which stimuli engage the brain’s motive systems, appetitive and defensive”* ([107], p.277).

The results of this ancillary behavioral experiment revealed that *Cottage Happy Shower* was rated as the more appetitive of the 8 FIPs used as stimuli (M_Pleasure_ = 5.92, SD = 1.75; M_Arousal_ = 5.97, SD = 2.19) and *Visior* the more neutral of the FIPs (M_Pleasure_ = 4.00, SD = 1.44; M_Arousal_ = 3.14, SD = 1.50). These two hygiene products were therefore selected for the fMRI experiment. We also selected a *Tetra Pak* fruit juice, the *Joker*, rated as appetitive (M_Pleasure_ = 6.50, SD = 1.36; M_Arousal_ = 5.93, SD = 1.98) and a *La Croix* bottle of bleach, referred to as Bleach, rated neutral (M_Pleasure_ = 3.03, SD = 1.66; M_Arousal_ = 2.63, SD = 1.51) as controls for the neuroimaging experiment.

Each one of these 4 products – like all other stimuli – was correctly categorized by the 52 participants during the debriefing of the ancillary experiment. *Cottage Happy Shower* belongs in the body care product category, *Visior* in the dish liquid one, *Joker* in the soft drinks one and bleach in the household cleaners one.

**Figure 1:**
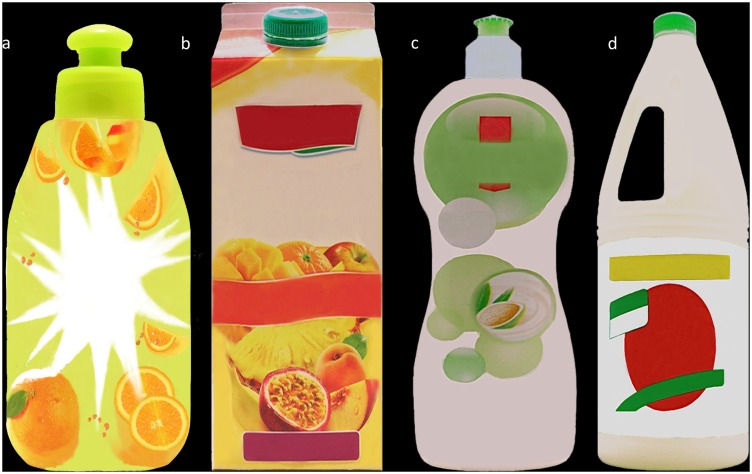
Visual stimuli in the fMRI experiment. (a) Standardized *Cottage Happy Shower Tequila Sunrise* (b) Standardized *Joker* fruit juice (c) Standardized *Visior* (d) Standardized bleach. (a–d) Stimuli were presented branded with the name Fabuloso but cannot be depicted as such in the article here due to the creative common license of PLOS ONE. Please follow this link for additional details on our stimuli and FIPs: http://fip.oullier.fr.

Our main hypothesis in the laboratory part of our work is that FIPs elicit non-conscious gustatory inferences in the brain of consumers. One of our regions of interest (ROIs) was the orbitofrontal cortex (OFC) known to be – among other things – the secondary gustatory cortex [12]. Given this region is very close to brain areas found to be participating in brand preferences on drinks (e.g., [108]), we standardized brand name on our stimuli using the term “Fabuloso”, after the product accidentally ingested in Texas and in the State of New York mentioned in the introduction of this article, given this brand name is unknown in France where our studies were conducted. We also removed information about the volume and the composition of the products to control for additional features that could have distracted the consumer but were not crucial for the purpose of this study (see Figure 1).

Four versions of each product were made by a horizontal inversion of half or the totality of the product label. All 16 stimuli were presented against a black background to minimize environmental cues [109] and improving visual contrast. Different product shapes were matched for proportionality [110] and pictures were modified for consistent size and resolution and to match the highest resolution (800×600 pixels) provided by the MRI compatible display setting available at the Marseille fMRI Center.

#### Task and fMRI block design

This experimental paradigm is a 1-back task in which participants were asked to monitor the identity of two consecutive stimuli and to indicate whether the currently presented stimulus is the same (or not) as the one presented during the previous trial. The attention of the participants is therefore focused on the stimuli. Hence possible brain activations related to gustatory inferences would be implicit to the visual discrimination task as it happens to be repeated in all conditions. The cerebral activity related to such a task is therefore theoretically controlled when contrasting our experimental conditions.

Four experimental conditions represent different product categories: a FIP that has been accidentally ingested mainly because of its package (*Cottage Happy Shower*), a FIP that has been accidentally ingested mainly because of its context (*Visior*), a soft drink (*Joker*) and a household cleaner (*Bleach*). Each condition is composed by the four different standardized visual versions of each product: e.g., the *Cottage Happy Shower* condition is composed of four versions of the *Cottage Happy Shower* product made by a horizontal inversion of half or the totality of the *Cottage Happy Shower* label. Participants performed the 1-back task on these versions of the *Cottage Happy Shower* product as stimuli within the *Cottage Happy Shower* condition.

**Figure 2:**
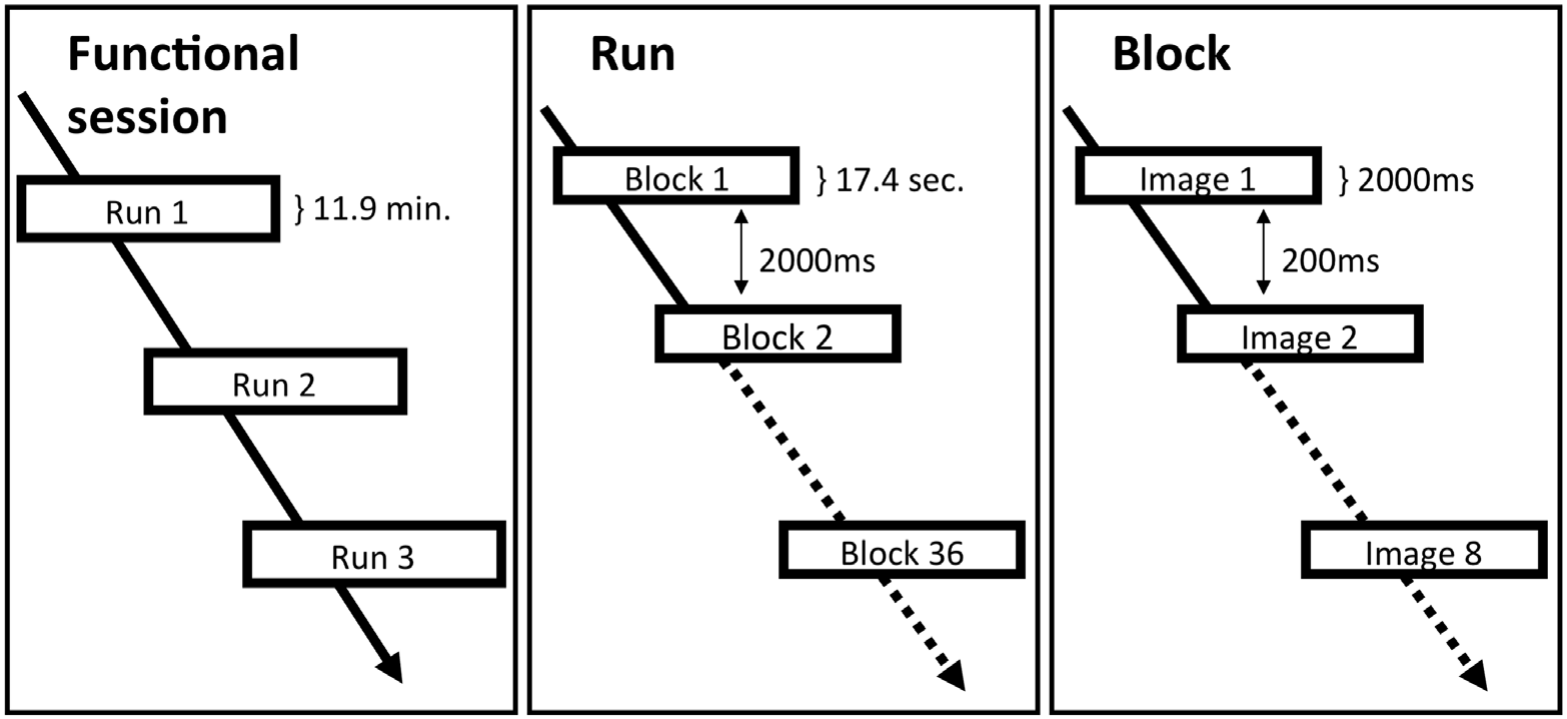
Block design of the fMRI experiment. A functional neuromaging session was divided in three functional runs randomized and counterbalanced across participants. Within each functional run, there was a total of 36 blocks of stimuli (9 blocks per condition, each of which contained 8 images). The interblock interval was 2000 ms. Within each block, stimulus presentation time was 2000 ms (with an interstimulus interval (ISI) of 200 ms) during which participants performed the 1-back task.

The whole functional session was divided into 3 functional runs randomized and counterbalanced across participants. Within each functional run, there was a total of 36 blocks of stimuli (9 blocks per condition, 8 stimuli per block). The interblock interval was 2000 ms (Figure 2). Within each block, stimulus presentation time was 2000 ms (with an interstimulus interval (ISI) of 200 ms) during which participants performed the 1-back task. Within each functional run, 63 responses were expected in each condition and, ideally, participants had to find 16 identical cases given that 25% of consecutive images within each functional run were the same.

#### Data acquisition and preprocessing

Functional neuroimaging data was acquired on a 3-Tesla BRUKER MEDSPEC 30/80 functional MRI scanner equipped with a circular polarized head coil. Whole brain anatomical MRI data was acquired using high-resolution structural T1-weighted image (MPRAGE sequence, resolution 1×1×1 mm) in the sagittal plane prior to the functional runs. A fieldmap acquisition (3D FLASH sequence inter-echo time 4.552 ms) was collected in order to estimate and correct the B0 inhomogeneity.

For functional neuroimaging, OFC being one of our region of interest, functional slices acquisition was axial oblique, angled −30° relative to AC-PC plane (e.g., [111]), in order to limit frontal distortions and to cover cortical and subcortical areas. This setting prevented us from collecting data at the cerebellar level. A T2*-weighted echo planar sequence was used with 30 interleaved 3 mm-thick/1 mm-gap slices (repetition time = 2000 ms, echo time = 30 ms, flip angle = 78,4°, field of view = 192 mm, 64×64 matrix of 3×3×3 mm voxels). Each participant performed 3 functional MRI runs. For each run, 357 functional volumes were acquired. The whole “in-scanner” part of the experiment lasted for about one hour (anatomical MRI and fieldmap acquisition included).

Six dummy scans in each run were discarded in order to ensure that the longitudinal relaxation time equilibration was achieved. Data was pre-processed and analyzed using SPM8 (Wellcome Department of Cognitive Neurology, London, UK). First, processing consisted in including the voxel displacement map computed using the fieldmap toolbox during the realign and unwarp procedure for distortion and motion correction. Second, the high-resolution structural T1-weighted image was coregistered to the mean EPI image. Third, all MRI volumes were processed with SPM8's New Segment option to generate grey matter (GM) and white matter (WM) images of the participants. Fourth, a DARTEL template was generated and (affine-only) spatial normalized to MNI space. Fifth, that DARTEL template was used to normalize functional data of each participant. Last, each participant’s normalized functional data was spatially smoothed using a 8 mm full-width at half isotropic Gaussian kernel.

Four regressors (one for each experimental condition: *Cottage Happy Shower*, *Joker*, *Visior* and Bleach) were modeled using a 2-second box-car waveform convolved with the canonical hemodynamic response function (HRF). To account for inter-subject variability in the group analysis, the contrast images obtained during the first level analysis were included in a second level t-test in order to conduct a random effects analysis at group level. A statistical significance threshold of *p*<.001 (uncorrected for multiple comparisons) and a spatial extent threshold of at least 3 contiguous voxels was used in the random effects analyses. Images resulting from these analyses were inclusively masked using *a priori* regions of interest defined by the WFU Pickatlas software [112] and chosen after the functional neuroimaging literature on visual food processing [82]. These regions included the fusiform gyrus, the rolandic operculum, the insula and the frontal lobe’s lateral (dorsolateral superior frontal gyrus, middle frontal gyrus, opercular part inferior frontal gyrus, triangular part inferior frontal gyrus) and orbital surfaces (orbital part superior frontal gyrus, medial orbital superior frontal gyrus, orbital part middle frontal gyrus, orbital part inferior frontal gyrus). Regions of interest were anatomically defined using the automated anatomical labeling (AAL) software [113] and have been 1-voxel dilated by using the 2D dilation function. The anatomical localization of the activations was provided by the MNI stereotaxic space and the neuroanatomical labeling of significant foci performed using AAL.

Given that the *Cottage Happy Shower* had been ingested because of a confusion with orange juice and that we are looking for implicit gustatory inferences triggered by this product, behavioral data on orange juice preferences for each participant collected before the fMRI experiment were used in the SPM8 random-effects multiple regression analysis on the contrast fMRI images in the processing of the *Cottage Happy Shower* condition. A statistical significance threshold of *p*<.001 (uncorrected for multiple comparisons) and a spatial extent threshold of at least 3 contiguous voxels was used in this multiple regression analysis within the defined ROIs. We controlled that any activity in the gustatory cortices in response to seeing *Cottage Happy Shower* was not related to explicit orange juice preferences by statistically regressing the participants’ orange juice preferences on BOLD activity. Indeed, as the hypothesized gustatory inferences are automatic and implicit when participants perform the 1-back task, it seems to us that these are mainly related to a categorization process rather than to participants’ preferences.

### Results

#### Behavioral results

The analysis of the behavioral dataset of the fMRI experiment (answers and reaction time inside scanner) was performed using SPSS (Version 17, SPSS Inc., Chicago, Illinois, USA). Given that we were interested in automatic and implicit gustatory inferences at the cerebral level [80], we tested that the 1-back task was performed similarly throughout the experimental session. The performances of participants in the 1-back task were therefore rated as percentages of inaccurate answer given in each condition. An inaccurate answer was defined as an unseen difference between two consecutive stimuli. Reaction times were relative to inaccurate answers. Comparisons were performed using the non-parametric Friedman test. When a significant statistical difference was found, the Wilcoxon signed-rank test (Bonferroni corrected for multiple comparisons) was employed.

For reaction times, there was no significant difference between the conditions across the sessions (N = 10; Chi-square = 1.971; d.f. = 3; *p* = .578; N<14 is due to the fact that 4 participants made 0 errors and therefore there were no reaction times whatsoever in their performance). A significant difference in accuracy of the answers across conditions was found in the 1-back task (N = 14; Chi-square = 12.429; d.f. = 3; *p<*.05). Given the Wilcoxon signed-rank test Bonferroni corrected for multiple comparisons (*p*<.0083), results indicated there was no significant statistical difference between the *Cottage Happy Shower*, *Joker* and *Visior* conditions – Bleach vs *Cottage Happy Shower* (Z = −2.731; *p* = .006) and Bleach vs *Joker* (Z = −2.668; *p* = .008). We will therefore only comment the neuroimaging results relative to these conditions.

The behavioral results following the fMRI session (i.e. outside the scanner) reveal that each participant had well identified and categorized the different stimuli (*Joker* as a fruit juice, *Cottage Happy Shower* as a shower gel, Visior and Bleach as household cleaners) and ate (therefore, confirming (s)he was hungry). Overall, it was found that *Joker* and *Cottage Happy Shower* were judged as more appealing than *Visior* and Bleach.

#### Neuroimaging results

**Figure 3:**
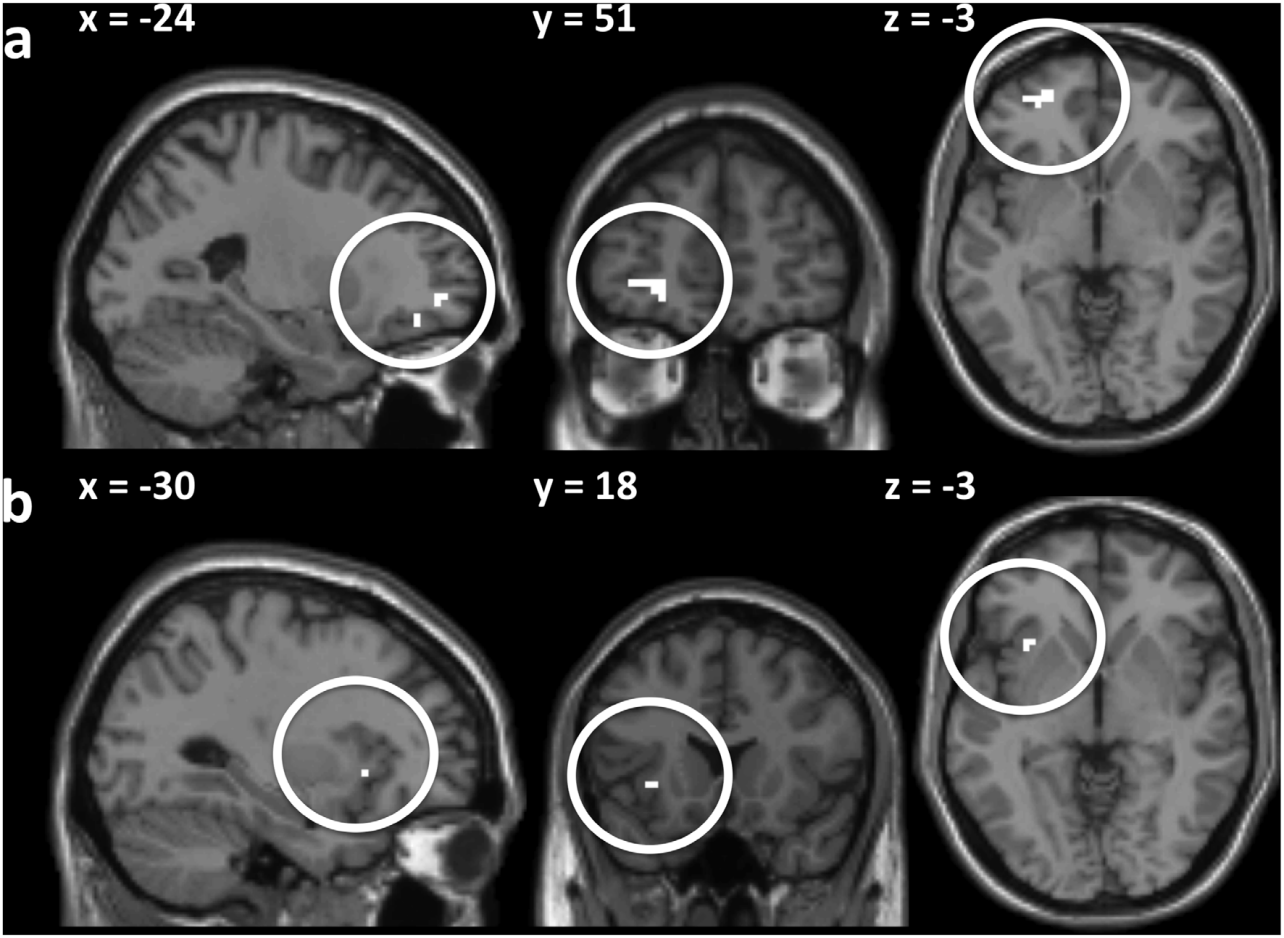
Main neuroimaging results. (a) Left orbitofrontal cortex activations (x = −24, y = 51, z = −3). (b) Left insular activations (x = −30, y = 18, z = −3). Statistical parametric maps of the *Cottage Happy Shower* vs *Visior* contrast (sagittal (x), coronal (y) and axial (z) views) (*p*<.001, uncorrected, cluster size >3 contiguous voxels) displaying the brain activity that is significantly higher when participants look at the *Cottage Happy Shower* compared to when they look at the *Visior.*

When comparing *Cottage Happy Shower* to *Visior*, we found a significant increase in activity in the insular cortex, the OFC and the fusiform gyrus (Figure 3). However, the *Joker* vs *Visior* contrasts revealed a significant increase in the activity of the the fusiform gyrus only at the chosen statistical threshold. The *Visior* related contrasts (*Visior* vs *Cottage Happy Shower* and *Visior* vs *Joker*) revealed no significant difference in brain activity (no suprathreshold clusters).

Detailed brain regions obtained by a random effect model showing significant activations (*p*<.001, uncorrected, cluster size >3 contiguous voxels) and labeled using AAL for the *Cottage Happy Shower* vs *Visior*, *Joker* vs *Visior*, *Visior* vs *Cottage Happy Shower* and *Visior* vs *Joker* contrasts are reported in Table 4 (see also Table S1 and Text S2 reporting the results of a whole brain analysis, i.e. significant activations (cluster size >3 contiguous voxels) surpassing a threshold of *p*<.001, uncorrected; the settings of acquisition of fMRI data did not allow to collect data in the cerebellum).

**Table 4:** Brain regions obtained by a random effect model showing significant activations (*p*<.001, uncorrected, cluster size>3 contiguous voxels) and labeled using AAL for the *Cottage Happy Shower* vs *Visior*, *Joker* vs *Visior*, *Visior* vs *Cottage Happy Shower* and *Visior* vs *Joker* contrasts (x, y and z refer to spatial coordinates in the MNI space).

			MNI coordinates (peak location)			Cluster size	
Contrast	Region	Lat	x	y	z	(in voxels)	T
**Cottage Happy Shower** **vs Visior**	Fusiform gyrus	R	27	−60	−9	36	8.72
	Fusiform gyrus	L	−27	60	−12	62	7.79
	Fusiform gyrus	R	36	−36	−24	25	7.48
	Fusiform gyrus	L	−36	−36	−21	28	5.83
	Superior frontal gyrus,orbital part (BA 10)	L	−24	51	−3	27	5.40
	Inferior frontal gyrus,orbital part	R	39	45	−3	4	4.64
	Middle frontal gyrus	R	39	33	27	6	4.39
	Insula (BA 13)	L	−30	18	−3	3	4.28
	Inferior frontal gyrus,triangular part	L	−48	27	27	4	4.13
**Joker vs Visior**	Fusiform gyrus	L	−24	−57	−12	53	6.08
	Fusiform gyrus	R	27	−57	−9	31	4.81
**Visior vs Cottage Happy** **Shower**	*No suprathreshold clusters*						
**Visior vs Joker**	*No suprathreshold clusters*						

The multiple regression analyses conducted on the contrast images of the *Cottage Happy Shower* vs *Visior* comparison revealed a significant relation between ratings of orange juice and BOLD activity only in the fusiform gyrus. Orange juice preferences were positively associated with increased activity in the bilateral fusiform gyrus (x = 42, y = −15, z = −24; T = 4.95, *p*<.001; x = −36, y = −6, z = −30, T = 4.31, *p*<.001) in the *Cottage Happy Shower* vs *Visior* contrast. No significant activation (no suprathreshold clusters) is negatively correlated on this contrast image.

## Discussion

The home poisoning and death cases reported earlier illustrate that FIPs have been found to be a specific category of mass consumption goods bearing potential danger for the health of the consumers [1], [49]. When asked, people explicitly mention that they would not drink a FIP intentionally, hence, cases of poisoning in the home as accidental poisonings are likely to be behavioral errors [37]. However, one cannot solely blame the consumer for making a mistake when the packaging of the product is the result of a highly elaborated and deliberate strategy aiming at using the visual codes of one category of product (foodstuffs) to enhance the consumer experience in another: the not so appealing hygiene and cleaning products. This does not mean at all that other factors but FIPs *per se* are not playing a role when people drink them. For instance, even if the danger FIPs represent is now undisputed in the public health sphere, in spite of most consumers not being aware of it because of little media coverage, several other factors could participate in the poisoning in the home, such as the state of mind of the consumer (e.g., distraction, stress, etc.) or the physical properties of the environment in which the poisoning is taking place. In other words, human and contextual factors are also at play and, to date, there is an obvious lack of scientific evidence that would help public authorities take firm(er) measures against FIPs. This is where our methodology and design, involving a combination of qualitative field study and quantitative laboratory experiment at several levels of analysis ranging from behavior to neurobiology can help. In a setting where the context and what the person does is controlled, and even when people are informed and aware that what they are going to see is not to be drunk, non-conscious taste inferences occur in their brains when they see a FIP. People would never explicitly say they would drink FIPs yet, implicitly, their brains are somewhat facilitating the drinking process as a confusion occurs because of a dangerous marketing strategy: packaging hygiene and cleansers as food products.

Following Loken et al. [114], we tested, in a consumer psychology perspective, the idea that FIP representations reside in modality-specific systems of the brain. We also considered real products involved in real life poisoning cases of real people. In doing so, we are in line with the grounded cognition view that *“laboratory paradigms could be more oriented toward explaining real-world phenomena”* ([115], p.324). Regarding the latter, our fMRI experiment was therefore designed to test data originating from both a field and a laboratory study. This is a clear departure from neuromarketing studies where, to date, products are, at best, tested before being launched on markets or, as it is the case in many studies, tested as such with little if no connection whatsoever to what people do with the product in real life (e.g., [116]–[119]).

As previously reported in several studies (e.g., [120], study 4, [121]), the design of our fMRI experiment aimed at a better understanding of cognitive mechanisms behind a specific consumer behavior. We appreciate whether a metaphor transfers experientially remote manipulations of bodily source concepts [122]. We suggest that food inferences elicited by the metaphor conveyed by the FIPs are mapped onto the target domain of hygiene products. In other words, the consequence of a food metaphor applied to a hygiene product is that implicit gustatory inferences can be found in the brain of the consumers. Such inferences most certainly participate in the accidental ingestion of a hygiene product.

As expected from the conceptual metaphor theory and the grounded cognition perspective, the *Cottage Happy Shower* versus *Visior* contrast revealed increased neural activations when looking at the former in all of our three regions of interest – insular cortex, OFC and fusiform gyrus. These areas have been repeatedly identified in the literature on visual food processing – reinforcing our belief that participants did implicit gustatory inferences while they were viewing this FIP, and the non-verbal metaphor it conveys. Moreover, except for the increased bilateral activity found in the fusiform gyrus, probably related to the emotional salience of the *Cottage Happy Shower* [123], we think these activations are not linked to the explicit preference participants exhibited for orange juice – as revealed in the post-experiment debriefing during which they identified the former product as a shower gel.

Because of a difference in the complexity of the Bleach stimulus revealed by pre-fMRI testing and given that we work on real world stimuli, in line with our behavioral responses inside the fMRI scanner, we only studied the *Joker* fruit juice versus *Visior* (FIP) contrast. The *Joker* fruit juice, when contrasted with the *Visior* revealed no significant increase of activity in the cerebral regions of interest we focused on, probably because of the lack of graspability of a product under that looks like a *Tetra Pak* package (see Text S2 for a discussion on the whole brain fMRI analyses).

Such a result leads us to the methodological concern that can be raised about our study, starting with the reverse inferences employed in interpreting the *Cottage Happy Shower* versus *Visior* contrast. There are reverse inferences when the engagement of a particular cognitive process is inferred from the activation of a particular brain region [124]. In our study, without a similar pattern of activations for the *Cottage Happy Shower* and the *Joker* on the same contrast, we inferred the existence of gustatory inferences from the co-activation of the insular cortex, OFC and fusiform gyrus. With no gustatory inferences when looking at the *Joker*, it is difficult to ensure that, in the sample population that participated in our fMRI experiment, the neural correlates of visual food cue processing are identical to the ones found in other studies relying on the same functional neuroimaging technique [80]–[82].

Moreover, even if the results of the Activation Likelihood Estimation (ALE) meta-analysis on the neural correlates of processing visual food cues [82] when contrasting food versus non food stimuli are very close to the brain's modality specific areas that Simmons et al. [80] found for food knowledge in the same kind of contrast images, only about 33% of the experiments included in the meta-analysis led to activations within the insular cortex, left lateral OFC and fusiform gyrus.

We nevertheless kept a bilateral activation hypothesis on these three regions of interest. First, because the meta-analysis is not only focused on studies with an implicit task such as Santel et al. [123] and Uher et al.’s [125] which could explain some activation differences. Second, in the Simmons et al. [80] study, lowering the cluster size threshold in their random effects analysis revealed a significant bilateral activity in the frontal operculum and in the OFC. Third, in the food versus non-food contrast image in Frank et al.’s ([81], supplemental table 3), 1-back task design revealed a significant bilateral activity in both the insular cortex and the lateral OFC. Regarding the latter, we do not follow the idea of a left lateralization of pleasantness in the OFC that was previously reported. For instance, in the Beaver et al. ([104], supplemental table 1) study, neural responses to images of appetizing food are localized in the left lateral OFC whereas the ones to images of disgusting stimuli are in the right lateral OFC. However, in the Schienle et al. ([126], table 2, supplemental table 2) study, there were bilateral OFC activations for pleasantness of high-caloric food as for disgust-inducing pictures.

We argue that the reverse inference methodological limitation is mitigated for the two following reasons.

First, even if a *small sample size undermines the reliability* of neuroimaging studies [127], our experimental design is similar to two other fMRI studies [80], [81], in which a random effects analysis can be found and allows for inferences to be made about such a population. Second, the fact the *Cottage Happy Shower* has been accidentally ingested by healthy adult consumers, withdrawn from the European market under the RAPEX procedure and rated on valence and arousal with an appetitive motivational dimension in our behavioral experiment, allows us to assume this product can lead to activations in gustatory cortices.

This calls for one more comment. The *Cottage Happy Shower* was a new product when it was accidentally ingested. And new products outweigh the categorizing difficulties [128], [129] that could explain poisoning (as miscategorization). Notwithstanding, the novelty of a hygiene product does not necessarily imply for it to look like food! Moreover, to our knowledge, all new hygiene products launched on the market are not ingested. Any new product can increase the risk of ingestion but its sole novelty is not sufficient to provoke the poisoning, the use of food metaphors is the key factor leading to such a consequence.

## Conclusion

The use of food metaphor in marketing strategies to improve the sales of hygiene product constitutes a serious health problem. One of the goals of the metaphoric content of a product package is to suggest the experience of product consumption (hygiene) in light of another experience (food). In this study, we built on a field study together with behavioral insights to design a final neuroimaging experiment aiming at investigating how (metaphoric) marketing strategies that led to FIPs induce confusion in the mind of the consumers. Here it happens to be the voluntarily evoked experience of food consumption for marketing purposes that leads people to drink shampoo. In other cases, it can be games [130], [131] such as relying on the so-called child-appealing products [3] inherited from toy packaging and metaphors (hygiene product is a toy) meant to associate washing dishes as something easy and fun (e.g., dishwashing is a piece of cake). Similarly to our study on FIPs, we strongly encourage public authorities to support scientific studies on this kind of novel metaphors.

Over the past 15 years, we have witnessed an increasing interest and use of neuroimaging techniques in the private sector to better understand consumer behavior [116]. More recently, several governments and governing bodies have been considering how behavioral and brain insights could inform policy making, when possible, particularly in health-related issues (e.g., [132]) and corporation-induced diseases [133] such as tobacco [134], obesity [135] and, in the following case, poisonings [136].

Our combination of a qualitative field study together with behavioral and neuroimaging data collected in a laboratory setting constitutes a potential useful benchmark for psychology and neuroscience to be used at the experimental level to inform health and many other domains of policy making [46]. We therefore believe that this work illustrates how “*ideas emerging in neuroscience* [*that*] *could potentially be a richer language for talking about cases like accidental child poisoning and, more broadly, about welfare and paternalism in some limited cases*” ([137], p.87). But, in our view, this implies to start from real cases in people’s real lives, to use multiple perspectives and levels of analysis to have a first qualitative analysis of these behaviors, then to design behavioral experiments that will allow to assess particular features. Finally, if some information cannot be revealed by the behavioral testing, relying on functional neuroimaging could lead to useful insights – without the use of brain sciences being mandatory as many private neuromarketing practices tend to promote [138].

Although we think our methodology constitutes an interesting benchmark for the use of behavioral and brain insights to inform policy making [46], we are well aware that it only covers one half of the process. For instance, providing qualitative data from real life health cases to narrow down the issue and identifying information only a functional MRI experiment can provide is without a doubt useful. But the other half of the process will require to use the ensemble of information and findings our results revealed in order to develop innovative and durable solutions to improve people’s health and well-being by implementing them. Then only would behaviorally and neuroscientifically evidence-informed policy make sense.

## Supporting Information

Figure S1 **Pictures of FIPs.**
(DOCX)

Table S1 **Additional fMRI results – Spatial localization of significant brain activity (whole brain analysis).**
(DOCX)

Text S1 **Pre-selection of FIPs – Ancillary behavioral experiment conducted prior to functional neuroimaging.**
(DOCX)

Text S2 **Additional fMRI results - Whole brain analysis.**
(DOCX)
