# Supplementary material for: Why People Drink Shampoo? Food Imitating Products Are Fooling Brains and Endangering Consumers for Marketing Purposes
Source: PLoS One. 2014 Sep 10;9(9):e100368. doi: 10.1371/journal.pone.0100368 (PMC4160172; doi:10.1371/journal.pone.0100368)
Supplement: Figure S1 — Pictures of FIPs. (DOCX) [file pone.0100368.s001.docx]

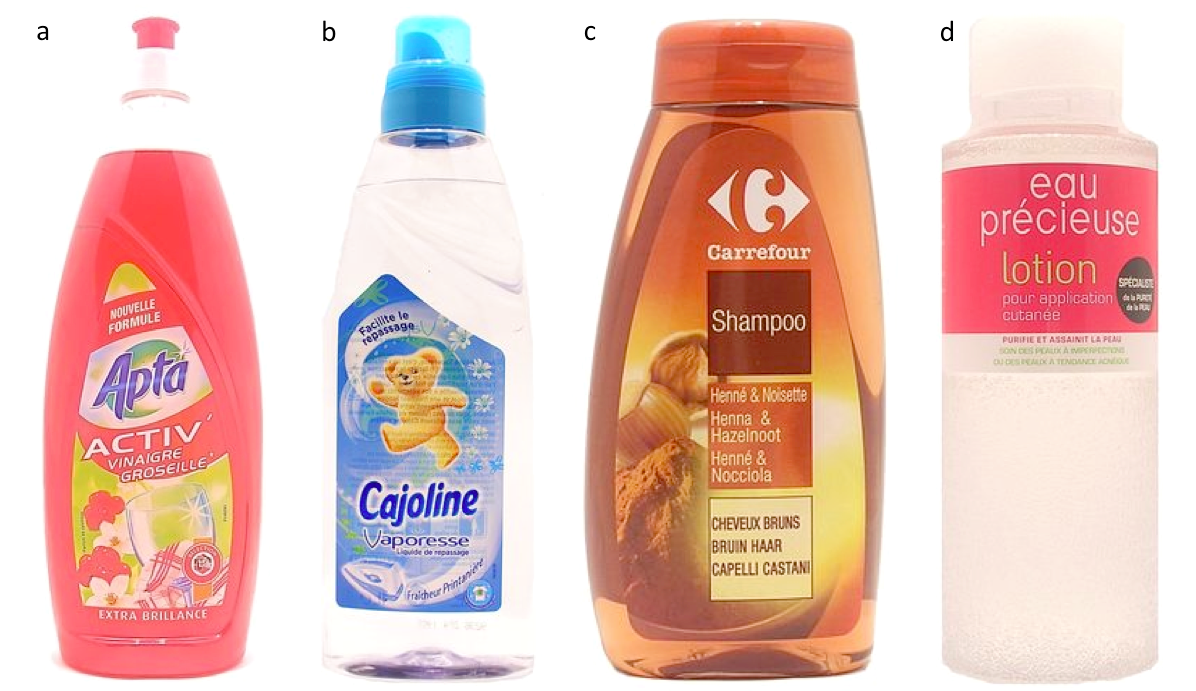


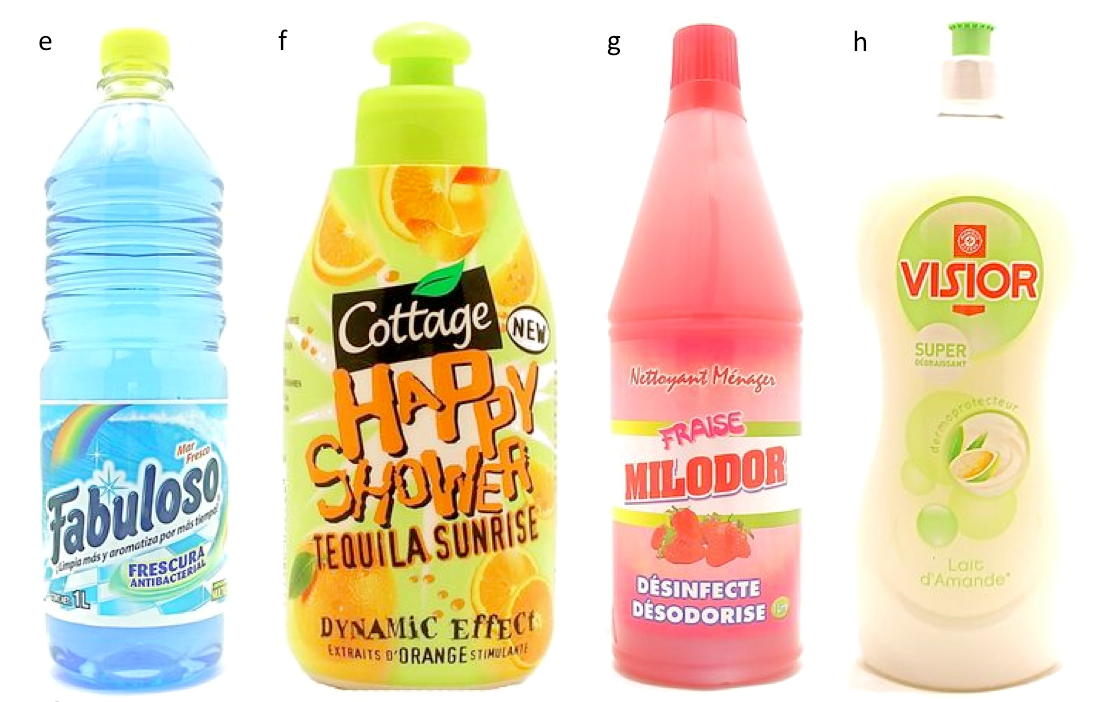


**Figure S1. Pictures of FIPs** (a) *Apta* berry vinegar. (b) *Cajoline Vaporesse*. (c) *Champion* henna and hazelnuts for brunettes. (d) *Eau Précieuse*. (e) *Fabuloso*. (f) *Cottage Happy Shower* Tequila Sunrise. (g) *Milodor* strawberry. (h) *Visior* sweet almond extract. (a-h) Pictures of the FIPs used in the ancillary behavioral experiment (test of appetitiveness).
