# Supplementary material for: Why People Drink Shampoo? Food Imitating Products Are Fooling Brains and Endangering Consumers for Marketing Purposes
Source: PLoS One. 2014 Sep 10;9(9):e100368. doi: 10.1371/journal.pone.0100368 (PMC4160172; doi:10.1371/journal.pone.0100368)
Supplement: Table S1 — Additional fMRI results – Spatial localization of significant brain activity (whole brain analysis). (DOCX) [file pone.0100368.s002.docx]

**Table S1** Additional fMRI results – Spatial localization of significant brain activity (whole brain analysis)

**Table S1.** Whole brain^1^ regions obtained by a random effect model showing significant activations (*p*< .001, uncorrected, cluster size > 3 contiguous voxels) and labeled using AAL for the *Cottage Happy Shower* vs *Visior*, *Joker* vs *Visior*, *Visior* vs *Cottage Happy Shower* and *Visior* vs *Joker* contrasts (x, y and z refer to spatial coordinates in the MNI space).

|  | | | **MNI coordinates** **(peak location)** | | | **Cluster size** |  |
| --- | --- | --- | --- | --- | --- | --- | --- |
| **Contrast** | **Region** | **Lat** | **x** | **y** | **z** | **(in voxels)** | **T** |
| **Cottage Happy Shower vs Visior** | Fusiform gyrus | R | 27 | -60 | -9 | 407 | 8.72 |
|  | Fusiform gyrus | R | 36 | -36 | -24 | 25 | 7.48 |
|  | Fusiform gyrus | L | -36 | -36 | -21 | 36 | 5.83 |
|  | Precuneus | L | -6 | -81 | 45 | 15 | 4.49 |
|  | Cuneus | L | -24 | -75 | 12 | 31 | 6.31 |
|  | Superior occipital gyrus | R | 18 | -84 | 30 | 8 | 4.41 |
|  | Superior frontal gyrus, medial part | R | 3 | 30 | 51 | 28 | 6.92 |
|  | Superior frontal gyrus, orbital part (BA 10) | L | -24 | 51 | -3 | 62 | 5.40 |
|  | Inferior frontal gyrus, orbital part | R | 39 | 45 | -3 | 4 | 4.64 |
|  | Inferior frontal gyrus, triangular part | L | -48 | 27 | 27 | 4 | 4.13 |
|  | Middle frontal gyrus | R | 39 | 33 | 27 | 6 | 4.39 |
|  | Middle frontal gyrus | R | 33 | 12 | 60 | 5 | 4.44 |
|  | Middle frontal gyrus | L | -33 | 6 | 51 | 5 | 5.18 |
|  | Middle frontal gyrus | L | -24 | 12 | 63 | 4 | 4.04 |
|  | Insula (BA 13) | L | -30 | 18 | -3 | 3 | 4.28 |
|  | Hippocampus | L | -21 | -27 | -6 | 7 | 5.18 |
| **Joker vs Visior** | Lingual gyrus | L | -24 | -57 | -12 | 53 | 6.08 |
|  | Lingual gyrus | R | 27 | -57 | -9 | 31 | 5.48 |
|  | Middle occipital gyrus | L | -27 | -75 | 12 | 3 | 4.52 |
|  | Superior frontal gyrus | R | 15 | 6 | 54 | 7 | 5.13 |
| **Visior vs Cottage Happy Shower** | Inferior temporal gyrus | L | -42 | 3 | -33 | 5 | 5.96 |
|  | Superior temporal gyrus | L | -45 | 0 | -12 | 4 | 4.96 |
|  | Temporal lobe (subcortical) | R | 27 | -45 | 12 | 14 | 4.86 |
|  | Temporal lobe (subcortical) | L | -27 | -45 | 18 | 5 | 4.80 |
|  | Brainstem | R | 15 | -15 | -18 | 7 | 4.75 |
| **Visior vs Joker** | Temporal pole | L | -39 | 12 | -30 | 34 | 6.60 |
|  | Anterior cingulate gyrus | R | 15 | 36 | 21 | 3 | 4.34 |

^1^ *Except cerebellum due to the fact that functional slices acquisition was axial oblique, angled -30° relative to AC-PC plane, in order to limit frontal distortions and to cover cortical and subcortical areas.*
