# Supplementary material for: Why People Drink Shampoo? Food Imitating Products Are Fooling Brains and Endangering Consumers for Marketing Purposes
Source: PLoS One. 2014 Sep 10;9(9):e100368. doi: 10.1371/journal.pone.0100368 (PMC4160172; doi:10.1371/journal.pone.0100368)
Supplement: Text S1 — Pre-selection of FIPs – Ancillary behavioral experiment conducted prior to functional neuroimaging. (DOCX) [file pone.0100368.s003.docx]

**Text S1** Pre-selection of FIPs – Ancillary behavioral experiment conducted prior to functional neuroimaging

In this ancillary experiment conducted prior to the neuroimaging one, 8 pictures of FIPs – the Fabuloso and 7 liquid hygiene products from our study of poisoning cases (Figure S1) – were presented amongst 24 pictures of liquid products: 8 liquid household cleaners, 8 liquid body care products and 8 soft drinks.

A pre-selection of the 8 FIPs was performed among the hygiene products to study.

To begin with, 3 solid hygiene products found in the qualitative study were excluded (Bleach tablet #B; Skip *Actigel* #O; Dishwasher tablet #S) from the set of visual stimuli to be used in the fMRI experimental setting because they could not be drunk. For the analysis, we just kept liquid hygiene products given that these products were ingested directly while they were still in their original package – i.e. the package that lead the consumer to pick them in the retail store.

Then, among the remaining 17 hygiene products, some were excluded from our set of visual stimuli to be used in the fMRI experimental setting because:

- either the hygiene product did not meet at least one of the criteria of the FIP Directive [4] (*Soupline* lavender #E),

- or the hygiene product was not considered by the patient herself (*O’Cedar* stratified and laminated flooring modern parquet #L), by the caller (*Visior* hygiene plus #C; *Le Comptoir de Famille* tomato leaf liquid soap #H; *Adidas* Sport Field menthol #M; *Soupline* #T) or by the control poison center physician (*Tropic force* multi-purpose household cleaner fruity atmosphere #G) as the main cause of the accidental poisoning,

- or the hygiene product was mistaken for a salted food product that is not of interest in the FIP marketing perspective (mayonnaise) (*Schwarzkopf Got2b* #R),

- or the hygiene product was ingested not because of its package but of a misuse (*Cosmence* #P),

- or the hygiene product belongs to a category (dish liquid) from which we had another product that was more relevant for our study (*Mir* vaisselle #I), because it was more clearly identified.

We selected 7 products from our qualitative study. All these hygiene products fulfilled at the least one of FIP criteria. Among these 7 products, 4 are household cleaners. Two were ingested in their context of use (*Cajoline Vaporesse* #J; *Apta* berry vinegar #Q), 1 out of its context of use (*Visior* sweet almond #A) and 1 in a home context with no additional information whatsoever (*Milodor* strawberry #K). The 3 others were body care products: 1 drunk in the context of its normal use (*Eau Précieuse* #N) and 2 out of such a context (*Champion* henna and hazelnuts for brunettes #D; *Cottage* *Happy Shower* Tequila Sunrise #F).

The 24 liquid products were selected after 129 individuals (females: N=76, males: N=53; age M = 20.4 ±1.36) answered unaided brand name recall questions.

All the color pictures of the products were taken with a digital camera, against the same white background, and standardized in size and definition (800×600 pixels). Before being used as stimuli in the functional neuroimaging experiment, these pictures were presented twice in a randomized order (Latin square design) to 52 individuals (females: N=27; males: N=25; age M = 22.1±0.32) and rated for valence and arousal [140] on a 9-point scale using the Self-Assessment Manikin (SAM [141]) thanks to a customized computer interface we designed in Labview (National Instruments, Austin, Texas, USA). Exposure duration to (unmasked) stimuli was 200ms. Each stimulus presentation was preceded by a 500ms fixation cross and followed by the presentation of both scales (5s each). The cursor appeared randomly on the scale to control for halo effects in the responses of participants. The main results of this experiment are reported in the body of the article.
