# Supplementary material for: Why People Drink Shampoo? Food Imitating Products Are Fooling Brains and Endangering Consumers for Marketing Purposes
Source: PLoS One. 2014 Sep 10;9(9):e100368. doi: 10.1371/journal.pone.0100368 (PMC4160172; doi:10.1371/journal.pone.0100368)
Supplement: Text S2 — Additional fMRI results - Whole brain analysis. (DOCX) [file pone.0100368.s004.docx]

**Text S2** Additional fMRI results - Whole brain analysis

In addition to the region of interest analysis of fMRI data provided in the main text, upon editorial suggestion, we also conducted a whole brain analysis (minus the cerebellum given the specific angle chosen for our fMRI data acquisition did not allow us to collect data in this region of the brain).

As reported in Table S1, when *Cottage Happy Shower* is compared to *Visior*, we found a significant increase in brain activity in the regions of interest we selected (insular cortex, OFC and fusiform gyrus) [82].

We also found activations in the frontal gyrus (left inferior frontal gyrus, bilateral middle frontal gyrus, right superior frontal gyrus), the occipital lobe (left cuneus, right superior occipital gyrus), the parietal lobe (left precuneus) and left hippocampus.

None of these activations resulting from the *Cottage Happy Shower* vs *Visior* contrast are significant at the *p*<.05 level with correction for multiple comparisons. However according to the literature on processing visual food cues, activations in the frontal gyrus (in the middle frontal and the superior frontal gyri) might contribute to the generation of food-related mental imagery [142]. This could suggest that increased activity in the frontal gyrus are related to its inhibitor role as participants identified *Cottage Happy Shower* as a hygiene product in spite of it looking like food, while being aware of the dangers that are related to its ingestion. Increased activity in the occipital lobe (left cuneus, right superior occipital gyrus) could result from the subjective salience of visual food cues in comparison with other non food-related objects [123]. Activations in parietal lobe (left precuneus) might be related to shifting attention between different locations in space [143], given subjects were asked to monitor differences between two consecutive stimuli. Hippocampal activations are common in visual food perception [103, 144-152]. The hippocampus is a brain region known for participating to long-term memory processes but also in reward [147]. According to Pelchat et al. [153], the hippocampus and the insula could participate in the sensory memory part of food desire – an assumption that makes sense in our study given the *Cottage Happy Shower*, since a metaphor re-enacts the experience of food. It appears that all of significant increases in brain activity that we found in this whole-brain analysis are in line with a lot of functional neuroimaging studies investigating the brain correlates of visual food perception.

As shown in Table S1, when comparing *Joker* fruit juice to *Visior*, we did not find a significant increase in activity in the insular cortex, the OFC and the fusiform gyrus, as expected. However, we found increased activity in the right superior frontal gyrus and the occipital lobe (left middle occipital gyrus and bilateral lingual gyrus). None of these activations when contrasting *Joker* vs *Visior* are significant at the *p*<.05 level (with correction for multiple comparisons). As for the increased activity in the frontal gyrus, contrary to *Cottage Happy Shower*, it might be difficult to suggest that these activations are related to its inhibitor role, given participants have all identified *Joker* as fruit juice. Yet, this frontal activity comes in addition to occipital one (mainly in the left lingual gyrus) that extends to the parahippocampal and fusiform gyri, both reported to participate in the perception of visual food cues. To some extent, activations found in the *Joker* vs *Visior* contrast might be related to gustatory imagery [142]. Moreover, lingual activations are related to the perception of visual food cues because of their subjective salience [123].

As shown in Table S1, when comparing *Visior* to *Cottage Happy Shower*, we found significant differences in activity located in the temporal gyrus (left superior and left inferior), the bilateral subcortical temporal lobe and the right brainstem. In the literature on a visual food perception, temporal activations from contrasts of non-food vs food stimuli are interpreted as related to object perception [80, 154]. We have no specific interpretations regarding the ones in the brainstem. However, given the bilateral subcortical temporal lobe activations related to this *Visior* vs *Cottage Happy Shower* contrast, some of them are located in the cerebrospinal fluid, there might be false positive activations that call for post hoc corrections (p<.05). In the following case, this contrast reveals no increased activity.

As shown in Table S1, when comparing *Visior* to *Joker* fruit juice, we found a significant increase in activity in the temporal pole (left middle temporal gyrus) and the anterior cingulate gyrus. We know from our ancillary experiment (Text S1), and from the debriefing conducted with participants after completion of the fMRI session that *Visior* is rated as less pleasant than *Joker* which might explain why this pattern of activations is located in regions that are part of the limbic lobe. Moreover, this pattern being strictly different from the brain activations of the *Visior* vs *Cottage Happy Shower* contrast, it seems that, first, the activations related to the *Visior* are inconsistent from one contrast to another and, second, radically different from activations expected from food (or from a food metaphor).
